# INTEGRATION Large-Scale Modeling Framework of Direct Cellular Vehicle-to-All (C-V2X) Applications

**DOI:** 10.3390/s21062127

**Published:** 2021-03-18

**Authors:** Mohamed M. G. Farag, Hesham A. Rakha, Emadeldin A. Mazied, Jayanthi Rao

**Affiliations:** 1 Center for Sustainable Mobility, Virginia Tech Transportation Institute, Virginia Tech, Blacksburg, VA 24061, USA; mmagdy@aast.edu (M.M.G.F.); emazied@vt.edu (E.A.M.); 2 College of Computing and Information Technology, Arab Academy for Science, Technology, and Maritime Transport, Alexandria 21500, Egypt; 3 The Charles E. Via, Jr. Department of Civil and Environmental Engineering, Virginia Tech, Blacksburg, VA 24061, USA; 4 The Bradley Department of Electrical and Computer Engineering, Virginia Tech, Blacksburg, VA 24061, USA; 5 Electrical Engineering, Sohag University, New Sohag, Sohag 82511, Egypt; 6 Ford Motor Company, Dearborn, MI 48126, USA; jrao1@ford.com

**Keywords:** connected vehicles, C-V2X, V2V, INTEGRATION software, traffic simulation, communication modeling

## Abstract

The transportation system has evolved into a complex cyber-physical system with the introduction of wireless communication and the emergence of connected travelers and connected automated vehicles. Such applications create an urgent need to develop high-fidelity transportation modeling tools that capture the mutual interaction of the communication and transportation systems. This paper addresses this need by developing a high-fidelity, large-scale dynamic and integrated traffic and direct cellullar vehicle-to-vehicle and vehicle-to-infrastructure (collectively known as V2X) modeling tool. The unique contributions of this work are (1) we developed a scalable implementation of the analytical communication model that captures packet movement at the millisecond level; (2) we coupled the communication and traffic simulation models in real-time to develop a fully integrated dynamic connected vehicle modeling tool; and (3) we developed scalable approaches that adjust the frequency of model coupling depending on the number of concurrent vehicles in the network. The proposed scalable modeling framework is demonstrated by running on the Los Angeles downtown network considering the morning peak hour traffic demand (145,000 vehicles), running faster than real-time on a regular personal computer (1.5 h to run 1.86 h of simulation time). Spatiotemporal estimates of packet delivery ratios for downtown Los Angeles are presented. This novel modeling framework provides a breakthrough in the development of urgently needed tools for large-scale testing of direct (C-V2X) enabled applications.

## 1. Introduction

As part of the 3rd Generation Partnership Project (3GPP) Release 14, Cellular Vehcile to everything (C-V2X) defines two transmission modes that together enable a broad range of use cases. Direct C-V2X, which includes vehicle-to-vehicle (V2V), vehicle-to-infrastructure (V2I), and vehicle-to-pedestrian (V2P) communication, provides enhanced range and reliability in the dedicated Intelligent Transportation System (ITS) 5.9-GHz spectrum that is independent of the cellular network, as well as network communications (V2N) in the traditional mobile broadband licensed spectrum. When compared with 802.11p-based technologies, Direct C-V2X provides increased communication range, better nonline-of-sight performance, enhanced reliability, and cost efficiency without relying on cellular network infrastructure assistance or coverage for enhanced safety services [1].

The modeling of Connected Vehicle (CV) and connected automated vehicle (CAV) applications necessitates coupling traffic and communication modeling tools to develop a fully integrated and dynamic modeling framework that captures the interdependencies of both the transportation and communication systems. A number of attempts have been undertaken to achieve this goal. However, current communication tools are incredibly slow and not scalable [2]. Consequently, there is an urgent need to develop scalable integrated traffic and communication tools. The proposed effort addresses this urgent need by developing an integrated traffic and communication modeling tool that captures the mutual interactions of both systems.

The main goal of this work is to develop a state-of-the-art scalable modeling tool that captures the mutual interaction of the communication (analytical C-V2X model) and transportation (INTEGRATION) systems and is capable of modeling hundreds of thousands of vehicles. The main contributions of this article are:

1 We propose a highly scalable bi-directionally coupled integrated simulator that incorporates the analytical model of C-V2X LTE-V release 14 communication standard in the INTEGRATION traffic simulator.
2 In the proposed integrated simulator, we used the spatial notion of density (see Section 5.3) for calculating the traffic density, which is a generalization of the linear notion of traffic density. We generalize the assumption in the analytical model [3] where vehicles are equally spaced and the traffic density is fixed in the highway scenario (as density was assumed to be the inverse of the inter-vehicle distance).
3 We developed a scalable implementation by applying the techniques of vectorization and precomputations of different values used in the calculation of the Packet Delivery Ratio (PDR) at predefined distances, using FORTRAN programming language which is much faster than Matlab (the original implementation).
4 We further enhance the computational time by utilizing a highly scalable, accurate, and efficient spatial index for two important tasks: (1) storing, retrieving, and updating vehicle positions, and (2) searching for vehicles around a given point within a specified range. The spatial index allowed us to track, during the simulation, the position of hundreds of thousands of vehicles at a 10 Hz frequency. In addition, using the spatial index, we managed to find only the vehicles that will be in communication range and could receive the message of a transmitting vehicle. Thus, the PDR calculations are computed for those vehicles only, instead of all vehicles in the road network.

In order to achieve these contributions, we made a number of trade-offs. In the first trade-off, we considered, for the sake of scalability, using an analytical model to abstract the communication system instead of using a communication network simulator. Although an analytical model is not as accurate or as detailed as a network simulator, the analytical model we used performed very well in comparison to the network simulator as was demonstrated in [3]. In our second trade-off, we made temporal and spatial assumptions regarding the PDR calculations. In the temporal assumption, we assumed that the PDR values do not change over a dynamic update interval that increases in duration as the level of traffic congestion increases (and hence need not be re-calculated). The update interval is calculated and changed dynamically during the simulation ranging between one second (no congestion) and seconds (in high congestion). In the spatial assumption, we assumed that, for a transmitting vehicle, not all vehicles in the road network would receive its message. Only vehicles within a specific range may be able to receive the message based on their corresponding PDR value.

With regard to the paper layout, Section 2 describes the related work while Section 3 describes the analytical communication model used in the proposed framework. Section 4 describes the characteristics of the traffic simulator used in the proposed integrated framework. Section 5 describes the architecture and methodology of the developed integrated simulator and the bidirectional coupling of the traffic and communication models. Section 6 presents the results of testing the proposed integrated simulator. Section 7 presents the conclusions of the paper and potential future work.

## 2. Related Work

**Figure 1:**
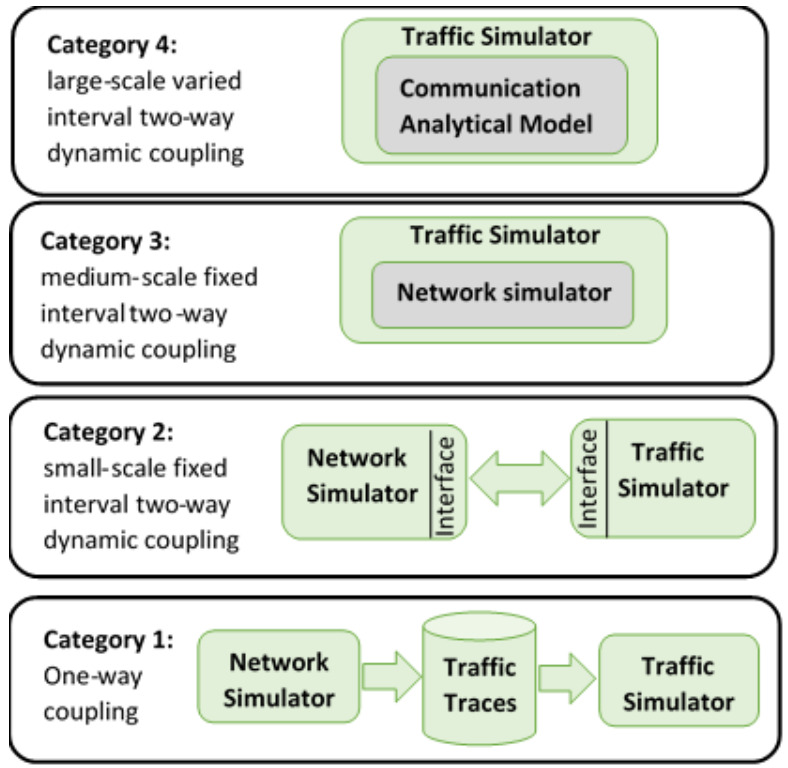
Integrated simulator categories.

We can categorize the previous work on integrating traffic and communication network simulators into four categories, as illustrated in Figure 1. In the first category, the two simulators are loosely integrated offline. Specifically, the traffic simulator is first run for the required mobility scenario generating the corresponding vehicle traces. Subsequently, the communication network simulator then reads these vehicle traces and applies the communication scenarios on top of these traces. While this approach allows for flexibility, it is also very limited in terms of the possible interactions between the two simulators. For example, it cannot be used to study the impact of the communication system on the transportation system performance given that the interaction is only one-way.

Most of the recent work on integrating the traffic and communication simulators lies in the second category. Veins [4], one of the best known frameworks in this category, integrates SUMO, a traffic simulator, and OMNET++, a communication simulator, using the TraCI interface. TraCI is a messaging standard that uses the Transmission Control Protocol (TCP) connections to share messages across the two simulators. One advantage of using TraCI is that it allows for bidirectional coupling of the two simulators. However, a key shortcoming of Veins is that it is unable to model large-scale networks. Veins by default do not support C-V2X mode 4; however, OpenCV2X [5] has extended Veins to support C-V2X modeling. Specifically, OpenCV2X implemented the C-V2X standard in SimuLTE, which is built on top of OMNET++ to support LTE communication.

Another Category 2 integrated simulator was developed in [6], which entailed integrating the SUMO traffic and NS-3 communication simulators. The authors extended NS-3 to support C-V2X release 14. The authors did not mention how the integration between NS-3 and SUMO was done, but we assume that they used the TraCI interface as was done in Veins because TraCI is the external interface for SUMO developed by SUMO authors.

Another second category system was developed in [2,7]. In this system, the authors developed an integrated simulator, VNetIntSim, which combined the OPNet communication simulator with the INTEGRATION traffic simulator. The integrated framework was used to demonstrate the impact of mobility parameters (traffic stream speed and density) on the communication performance through different applications including the File Transfer Protocol (FTP) using TCP and Voice over Internet Protocol (VoIP) based on the User Datagram Protocol (UDP).

A different variant of Category 2 simulators attempts to address the scalability problem by parallelizing one or both simulators to speed up the execution time and thus support simulation of large-scale road networks with hundreds of thousands of vehicles. In [8], the authors developed an Integrated Distributed Connected Vehicle Simulator (IDCVS) by incorporating hardware-in-the-loop simulation with the integration of SUMO and OMNET++. The authors provided a partitioning heuristic algorithm that partitions the complex traffic network into two sets of partitions, one for SUMO and one for OMNET++. The tool was then used to model Dedicated Short Range Communication (DSRC) communication for connected vehicles considering different levels of market penetration.

Vehicular Network Simulator (VNS) [9] integrates the traffic simulator DIVERT 2.0 and the network simulator, NS-3, falls into the third category of methods. VNS supports the 802.11p communication standard as it was developed before the release of the C-V2X LTE standard. VNS differs from the previous work we discussed in the way that NS-3 and DIVERT are integrated. Since both simulators are developed in the same programming language (C++), they were put into one executable environment instead of having the two executing programs communicating with each other. Although the two simulators share the same execution environment, they still communicate using TCP connections through the network integration module. In VNS, at each simulation time step, the traffic simulator is run first followed by the communication simulator. The communication simulator has node entities that are mapped to the vehicle entities in the traffic simulator. Each node entity has access to its corresponding vehicle entity. One last difference in VNS is the adaptation of the NS-3 network simulator. The authors adapted the implementation of NS-3 to support large-scale simulations. They applied the concept of nearest neighbors and the locality of vehicle position updates by using QuadTrees to accelerate the performance of the NS-3 communication simulator. However, the integrated simulator is still constrained by the computational speed of NS-3.

In the fourth category, similar to the third category, the network simulator is incorporated in the traffic simulator. However, in the fourth category, the network simulator is abstracted using an analytical model instead of using a network simulator. In [10,11], the authors developed an analytical communication model for the Dedicated Short Range Communications (DSRC) Media Access Control (MAC) layer protocol that estimates the packet drop probability and delay using a Markov chain and a queuing model. They integrated the analytical model into the traffic simulator INTEGRATION and tested the integrated framework using a dynamic eco-routing application.

Table 1 summarizes the various integrated simulators reported in the literature and shows how our proposed integrated simulator advances the state-of-art. In Table 2, we describe the different road networks, simulation times, number of vehicles, and execution time for the various integrated simulators. Table 2 clearly shows that our integrated simulator can efficiently support large-scale simulations.

Our work falls into the fourth category where we incorporate the analytical model of the C-V2X Release 14 protocol in the INTEGRATION traffic simulator. Similar to VNS in the third category, we enhance the computation time of large scale simulations by adding capabilities for finding vehicles within communication and interference range and indexing vehicles’ positions into a spatial index data structure. We opted to use the Grid Cells data structure as it is computationally more efficient than QuadTrees, which was used in VNS. We also enhanced the update operation of the vehicle positions as will be explained in Section 5. Another abstraction that we developed entails the dynamic coupling of the network and traffic modules. In our work, the traffic simulator uses the C-V2X analytical model at varying intervals instead of fixed intervals. The intervals are dynamically computed based on the congestion level of the road network, thus saving computational time and allowing for large-scale simulations, as will be explained in Section 5.

## 3. Analytical Communication Model

In this paper, we use the analytical communication model developed in [3]. This model abstracts C-V2X LTE release 14 [12]. In [3] the model was used to calculate the PDR values using the mobility patterns described in the 3GPP specification and was validated against Veins [13]. We summarize the analytical model here for clarity; however, the derivation and full details of the model can be found in [3].

The packet delivery ratio (PDR) is the probability that a message sent from a transmitting vehicle is received by a receiving vehicle within the transmitting vehicle’s communication range. The analytical model abstracts the PDR as the probability that none of four errors occurs during the transmitting and receiving operations. The four errors capture the different issues a packet may encounter that may result in the packet either not being received or being received in a damaged form that makes it impossible for the message to be decoded by the receiver. Equation (1) shows the relation between the PDR and the four possible errors:

(1) $$PDR=\left(1-E_{HD}\right)\cdot \left(1-E_{SEN}\right)\cdot \left(1-E_{PRO}\right)\cdot \left(1-E_{COL}\right),$$

where

$E_{HD}$ = the half duplex channel error probability,

$E_{SEN}$ = the power signal sensing error probability,

$E_{PRO}$ = the propagation error probability,

$E_{COL}$ = the collision error probability.

The packet is correctly received (with probability = PDR) if none of these errors occurs.

The half duplex error captures the probability that two communicating vehicles send packets in the same time frame, which is modeled as:

(2) $$E_{HD}=\frac{\lambda }{1000},$$

where λ is the packet generation rate in packets/s and 1000 is the number of millisecond time frames in a second. Consequently, this error models down to the sub-frame level.

The sensing error represents the probability that the received signal is lower than the power sensing threshold and thus the signal cannot be sensed, preventing the packet from being received. The sensing error is modeled as:

(3) $$E_{SEN}=\frac{1}{2}\left(1-\mathrm{erf}\left(P_{t}-PL\left(d_{t,r}\right)-P_{SEN}\right)\right),$$

where erf is the error function, $P_{t}$ is the signal transmitting power, *PL* is the path loss function, $d_{t,r}$ is the distance between the transmitting vehicle *t* and receiving vehicle *r*, and $P_{SEN}$ is the signal power sensing threshold. The only variable the sensing error function requires from the traffic simulator is the distance between the transmitting and receiving vehicles. This distance varies dynamically as vehicles travel in the network.

The propagation error represents the probability that a packet is received with a signal power larger than the power sensing threshold but the signal-to-noise ratio (SNR) is very low, making it impossible to decode the signal, i.e., the signal is distorted and cannot be understood because of the noise and thus the packet is considered not received. The propagation error is modeled as:

(4) $$E_{PRO}=\sum_{s=-\infty }^{\infty }BL\left(s\right)\times f_{SNR|P_{r}>P_{SEN},d_{t,r}}\left(s\right),$$

where BL(s) is the block level error (lookup table) at SNR equal to *s* and $f_{SNR|P_{r}>P_{SEN},d_{t,r}}\left(s\right)$ is the probability distribution function of the SNR random variable. It represents the probability of having an SNR with a value equal to *s* given that the power of the received signal exceeds the power sensing threshold and is modeled as:

(5) $$f_{SNR|P_{r}>P_{SEN},d_{t,r}}\left(s\right)=\left\{\begin{array}{cc} \frac{f_{SNR,d_{t,r}}\left(s\right)}{1-E_{SEN}}, & \mathrm{if}\;P_{r}\geq P_{SEN}\\ 0, & \mathrm{otherwise} \end{array}\right.$$

Finally, the collision error represents the probability that a packet is lost (not received) due to interference generated from an interfering vehicle transmitting a packet using the same resources at the same instant in time that the transmitting vehicle sends its message. Modeling the collision error is complicated. We try to summarize the final representation here; however, full details can be found in [3]. The collision error is modeled as:

(6) $$E_{COL}=1-\prod_{i}\left(1-E_{COL}^{i}\left(d_{t,r},d_{t,i},d_{i,r}\right)\right)$$

where

(7) $$E_{COL}^{i}\left(d_{t,r},d_{t,i},d_{i,r}\right)=p_{SIM}\left(d_{t,i}\right)\times p_{INT}\left(d_{t,r},d_{i,r}\right)$$

$p_{SIM}$ is the probability of two vehicles transmitting simultaneously. This probability can be calculated as:

(8) $$p_{SIM}=\alpha \;p_{SIM}^{2}\left(d_{t,i}\right)+\left(1-\alpha \right)\;p_{SIM}^{3}\left(d_{t,i}\right)$$

and $p_{INT}$ is the probability of interference and can be calculated as:

(9) $$p_{INT}\left(d_{t,r},d_{i,r}\right)=\frac{p_{SINR}\left(d_{t,r},d_{i,r}\right)-E_{PRO}\left(d_{t,r}\right)}{1-E_{PRO}\left(d_{t,r}\right)}$$

where

(10) $$p_{SINR}\left(d_{t,r},d_{i,r}\right)=\sum_{s=-\infty }^{\infty }BL\left(s\right)\times f_{SINR|P_{r}>P_{SEN}}\left(s\right)$$

$f_{SINR|P_{r}>P_{SEN}}\left(s\right)$ is the probability distribution function of the received signal power at the distances $d_{t,r}$ and $d_{i,r}$.

The collision error calculations depend on the identification of interfering vehicles. Subsequently, the probability of interference for each interfering vehicle is calculated as ($E_{COL}^{i}\left(d_{t,r},d_{t,i},d_{i,r}\right)$). The collision error is one minus the probability of having no interference for all interfering vehicles. The collision error for any of the interfering vehicles depends on three distances: the distance between the transmitting and receiving vehicles, the distance between the transmitting and interfering vehicles, and the distance between the interfering and receiving vehicles.

The probability of having a packet collision at a receiving vehicle depends on having a transmitting and interfering vehicle transmitting packets simultaneously and the two signals interfering with each other, leading to the received signal being distorted and unable to be decoded by the receiving vehicle.

The probability of two signals interfering with each other is modeled considering the interfering signal as a noise to the transmitting signal and thus the signal to interference and noise ratio (SINR) random variable is used to represent this probability and is modeled similar to the SNR random variable in the $E_{PRO}$ calculations.

The probability that the transmitting and interfering vehicles simultaneously send their packets using the same resources is attributed to the fact that the two vehicles cannot sense each other during the scheduling process. This process depends on the last two steps of the sensing-based semi-persistent scheduling algorithm (SB-SPS). The authors of the analytical model divided the modeling process of this probability into two mutually exclusive probabilities (step 2 and step 3 in the sensing-based semi-persistent scheduling, where in step 2 we select at least 20% of the available resources as assignable resources, and this step is only used in the case of high channel busy ratio (CBR), while, in step 3, we select from the assignable resources exactly 20% of the available resources, and this step is used only in the case of low channel load (low CBR), for details of the scheduling algorithm, check the analytical model [3]). The two probabilities are combined by an experimentally configured variable α. The value of α was found to be equal to:

(11) $$\alpha =\left\{\begin{array}{cc} 0 & \;CBR\leq 0.2\\ 2\;CBR-0.4 & \;0.2<CBR\leq 0.7\\ 1\; & CBR>0.7, \end{array}\right.$$

where CBR is the channel busy ratio and is calculated during steps 2 or 3 of the scheduling scheme. The CBR calculations mainly depend on the distances between transmitting and receiving vehicles, receiving and interfering vehicles, and on the vehicles density on the road. The detailed modeling of the CBR can be found in the analytical model [3].

This model was validated against C-V2X implementation inside OMNET++ in VEINS and demonstrated to produce consistent results.

## 4. The INTEGRATION Traffic Simulator

### 4.1. Traffic Modeling Levels

Traffic behavior can be modeled at three levels: macroscopic, mesoscopic, and microscopic. Macroscopic modeling abstracts the traffic stream behavior as a compressible fluid. Alternatively, mesoscopic modeling typically entails tracking individual vehicles at an aggregate level of detail (i.e., not tracking the instantaneous longitudinal and lateral motion of a vehicle along a link). Finally, microscopic modeling entails replicating the lateral and longitudinal movement of individual vehicles at a detailed level of resolution capturing car-following and lane-changing behavior. The INTEGRATION traffic simulator [14,15] uses the microscopic modeling approach.

### 4.2. Simulator Basic Concept

INTEGRATION performs four main functions every 0.1 s. These functions are:

- **Vehicle Departures:** It creates individual vehicle trip departures based on an aggregate time-varying origin–destination (OD) matrix.
- **Traffic Assignment:** It moves vehicles onto subsequent links using ten different traffic assignment methods. Traffic assignment establishes the chain of links that connects the trip origin to its destination while balancing traffic congestion in the network.
- **Longitudinal Vehicle Motion:** It moves vehicles along links based on desired speeds for each candidate distance headway tempered by prevailing vehicle dynamics and collision avoidance constraints that are only invoked when the lead vehicle is traveling slower than the following vehicle and the following vehicle is relatively close to the lead vehicle. Specifically, INTEGRATION uses the Rakha–Pasumarthy–Adjerid car-following model to capture the longitudinal movement of the vehicles [16]. Vehicle movement is constrained by a vehicle dynamic model described in [17].
- **Lateral Vehicle Motion:** It considers vehicle discretionary lane changing in selecting lanes that maximizes their speed. This model captures biases to stay on the rightmost lane and pass on the left. In mandatory lane changing, vehicles must be in specific lanes in order to follow their path and comply with lane restrictions [18].

Routing, lane-changing, and car-following logics are fully integrated where mandatory lane choices are driven by vehicle routing decisions and discretionary lane choices are driven by car-following desires.

The INTEGRATION model has been extensively tested and validated. For example, in [19], the INTEGRATION model estimates of vehicle delay were validated. In [20], the vehicle stop estimates are described and validated. The VT-Micro model [21,22] is used to estimate vehicle emissions and fuel consumption levels.

## 5. Integrated Simulator

### 5.1. Simulator Architecture

**Figure 2:**
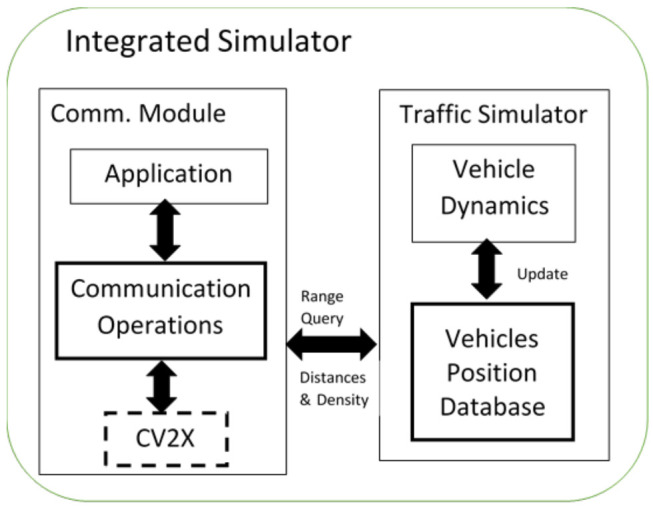
Integrated simulator architecture.

As was described in Section 2, the connection between the communication and traffic simulators can be categorized into four main categories: (a) one-way static coupling; (b) small-scale, two-way, fixed-interval dynamic coupling; (c) medium-scale, two-way, fixed-interval dynamic coupling; and (d) large-scale, two-way, variable-interval dynamic coupling. Our proposed approach is a first attempt at developing a large-scale, two-way, variable-interval dynamic coupling model. Specifically in this effort, we developed a fully integrated traffic and communication system, as illustrated in Figure 2. The traffic simulator models the motion of the vehicles and uses the vehicle’s position to identify the pairs of vehicles that are within communication and interference range. Using these dynamically updated positions, the PDR is computed and packets are either received or dropped depending on the computed PDR. The calculation of the probability of receiving a packet is repeated every 100 ms by generating a uniformly distributed random number between 0 and 1 and comparing it to the computed PDR. Packets with random numbers less than or equal to the PDR are received by the receiving vehicle while those larger are dropped. The packet generation rate, and, consequently, the PDR computation, are every 100 ms (10 times in a second). For efficient computations, we assumed the PDR values are the same for the duration of a second, i.e., the vehicle positions will have no or minimal changes during a second.

In this section, we describe these various coupling approaches and how our proposed model is unique.

In one-way static coupling, the traffic simulator is run first to generate traffic traces, which are then used by the communication simulator to model the vehicular communications. In the case of small-scale, two-way, fixed-interval dynamic coupling, a communication simulator is coupled with a traffic simulator through an intermediate layer (e.g., TraCI). In this setup, the traffic simulator acts as the server and the communication simulator acts as the client. The communication simulator sends/receives messages to/from the traffic simulator using the TraCI standard through TCP protocol. As such, TraCI acts as the intermediate layer that facilitates communication between both simulators at predefined, fixed-duration time steps. Unlike the first category, in the two-way, fixed-interval dynamic coupling, the two simulators are aware of each other and only the communication simulator uses the output of the traffic simulator.

The third category is medium-scale, two-way fixed-interval dynamic coupling, where the traffic simulator logic is incorporated in the communication simulator, hence removing the overhead of communicating between the two simulators using TraCI. This allows for scaling to larger simulations compared to the second category and provides for better two-way coupling between the two simulators. Still, the synchronization between the two simulators is done at predefined, fixed time steps (which is controlled by the simulator running at the higher frequency) and hence the entire simulation speed is affected by the slower simulator (which is the communication simulator as it runs at a millisecond scale while the traffic simulator, at best, is run at a decisecond scale). The fourth category is large-scale, two-way, variable-interval dynamic coupling. Unlike the third category, here we incorporate the communication simulator logic in the traffic simulator and replace the communication simulator with an analytical abstraction of the communication system. Using an analytical model together with a variable-interval, two-way interaction allows us to scale to very large road networks while minimally sacrificing the accuracy of calculations, thus providing a scalable modeling tool. Figure 1 shows the four categories and the relation between the network simulator and the traffic simulator in the four categories.

In our work, we opted to incorporate an analytical C-V2X communication model as a subroutine in the INTEGRATION traffic simulator. The subroutine is called at variable interval durations to allow for scalability. This approach enabled us to run large-scale simulations and fully support two-way dynamic coupling of the two simulators. To incorporate the analytical communication model in the INTEGRATION traffic simulator, we added four components to allow for seamless synchronization: a vehicle position database, a communication standard (C-V2X), a communication operator, and a communication application.

### 5.2. Vehicle Position Database

Traffic simulators step through time and track vehicle positions at fixed time intervals over the entire simulation. The communication application requires finding candidate vehicles within range of each other to identify V2V pairs. This is needed when querying the receiving vehicles for each transmitting vehicle and the interfering vehicles for each receiving vehicle. Assuming the communicating vehicles are equally distributed spatially, as was done in [3], is unrealistic. Instead, our approach is to dynamically compute and use the actual distances between vehicles on the road network to capture receiving and interfering vehicles.

Given the position of vehicles on the road network, we can easily find all vehicles within a certain range in the $O(n^{2})$ time by simply enumerating all pairs of distinct vehicles and computing the Euclidean distance between each pair. We then include only the pairs of vehicles with distances less than the specified range. This, however, is very slow in the case of large networks where the number of vehicles is huge since this process is done every time step for each vehicle in the road network during the entire simulation. Note here that we are assuming that there is a communication range beyond which the signal will be too weak and the packets will be lost. This assumption allows for the efficient computation of the PDR. The specified range is an input parameter to the simulator.

To support the calculation of the PDR in the analytical model, we need to store the vehicle positions in a data structure that facilitates scalable range queries and update operations. To achieve this objective, we utilized the idea of building a spatial index, an index that supports spatial data (*x* and *y* coordinates in our case). There are two main categories of spatial indices: file-based and memory-based approaches. Based on performance analysis done in [23], we used the memory-based approach for building our spatial index. The memory-based approach achieves approximately the same performance as the file-based approach but with less computational time, which is critical for our application.

**Figure 3:**
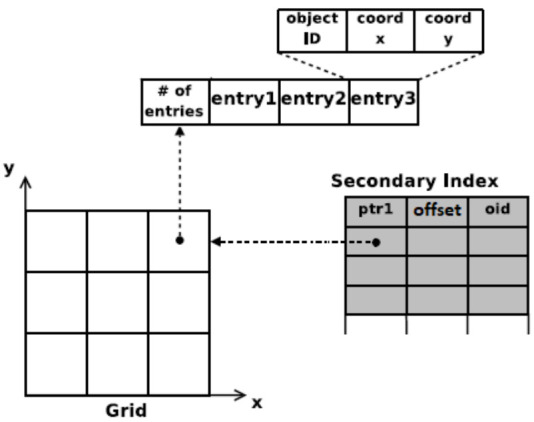
Grid-cells-based index, adapted from [23].

Grid cells is a memory-based approach for building spatial indices. The road network is divided into a grid of square cells of equal user-defined size. We maintain a two-dimensional (2D) array data structure to store the indices of the cells in the grid. Each vehicle’s position is indexed in the spatial index by calculating the cell coordinates it belongs to using the following equation:

(12) $$c\left(i,j\right)=\left\lfloor \frac{i}{m}\right\rfloor ,\left\lfloor \frac{j}{m}\right\rfloor$$

where *i* and *j* are the vehicle position coordinates and *m* is the cell size. Each cell maintains a list that stores all the information about the vehicles that are currently indexed in it. The information stored about each vehicle is the vehicle ID and the vehicle position (*i* and *j* coordinates). The spatial index here is represented by the 2D array and the list in each element of the 2D array. Using these two data structures, we can know for each cell in the grid the set of vehicles that are indexed in it. Using the spatial index, we are able to map from the cells to the vehicles, i.e., for each cell, we can determine the set of vehicles in each cell. In order to do the inverse operation (i.e., for each vehicle, we need to know its place in the cell list), we utilize another data structure that allows this inverse operation. Mainly, we use another array of size equal to the number of vehicles in the network and store for each vehicle the cell number it belongs to and its place (offset) in the list of that cell. Using the two data structures mentioned, we have a two-way mapping between the vehicles and the cells. Figure 3 shows the two data structures used to form the grid cell spatial index. The two-way mapping is achieved by the two data structures, which provides a much more efficient update operation as shown and discussed in Section 5.2.2.

#### 5.2.1. Range Query

**Figure 4:**
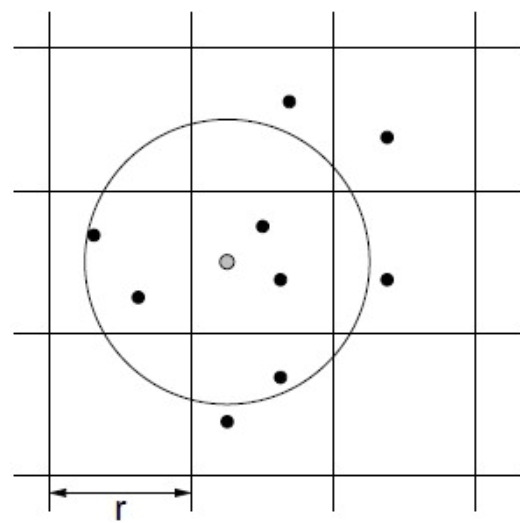
Grid cells.

One of the main tasks in calculating the PDR in the C-V2X analytical model is to calculate the distances between the transmitting vehicle and each of its receiving vehicles within the communication range (a configurable parameter as described earlier, e.g., 500 m) and also between a receiving vehicle and all the interfering vehicles around it within a predefined range (maximum distance at which a vehicle can cause interference with a receiving vehicle, a configurable parameter). Figure 4 shows the road network as a grid of cells. The query vehicle is the dot at the center of the circle in the figure. The other dots represent other vehicles in the network. The set of vehicles that lie within the circle are the ones that should be returned as a result of the range query. This task can be efficiently performed using the range query operation on the grid cells spatial index. A range query on a grid cell index requires to know the query cell coordinates and the range of the query described in terms of how many cells surrounding the query cell should be searched.

**Figure 5:**
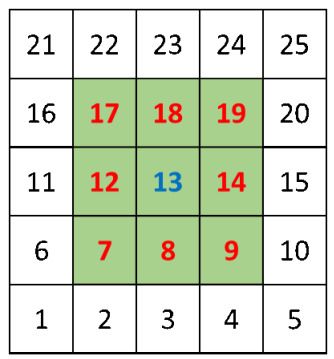
Range query in grid cells index.

For example, in the case of receiver–interferer vehicles, assuming a range of 1000 m around the query cell of the receiving vehicle in all four directions and assuming the cell size is 500 m, then the cells lying in the query range for a querying cell with coordinates (i,j) are the cells with coordinates in the range: (i−2,j−2) to (i+2,j+2). In general, the cells to be searched within the query range can be determined using the following equation:

From the cell with coordinates:

(13) $$\max\left(i-\frac{qr}{m},0\right),\;\max\left(j-\frac{qr}{m},0\right),$$

to the cell with coordinates:

(14) $$\min\left(i+\frac{qr}{m},i_{max}\right),\;\min\left(j+\frac{qr}{m},j_{max}\right),$$

where (i,j) are the coordinates of the querying cell, qr is the query range in meters, *m* is the cell size in meters, and $i_{max}$ and $j_{max}$ are the coordinates of the furthest cell in the grid. Thus, our task can be performed as a query range by simply finding the corresponding cell of the querying vehicle, then using the required distance (communication range in the case of sender–receiver and interference range in case of receiver–interferer) as the query range. The result of the query range operation is the set of vehicles that are at a distance less than or equal to the query range from the querying vehicle. The set is populated by iterating over the list of vehicles in each cell of the candidate cells to be searched and then calculating the distance between the querying vehicle and the vehicles in the list using the Euclidean distance. A vehicle is included in the result set if the calculated distance is less than the query range. Figure 5 shows the grid cell with the query vehicle in cell 13. The candidate cells to be searched are only the cells with red numbers besides the cell of the querying vehicle (the cell with number 13). Note here that we converted the cell coordinates (i,j) to just one number for simplicity of notation and implementation.

The grid cell spatial index allowed us to only have to search for vehicles in neighboring candidate cells instead of searching the entire set of vehicles in the network. The number of candidate cells to be searched depends on the query range distance and the size of the cell. The query range operation is thus performed in a time (nearly) linear as a function of *n* and *k*, O(n+k), where *k* is the number of candidate vehicles retrieved from the searched candidate cells, and *n* is the total number of vehicles in the network.

#### 5.2.2. Update Vehicle Positions

Updating the position of the vehicles during the simulation is done every Δ*t*, where *t* is defined by the user of the simulator. The mobility application updates its state every decisecond, while the communication application updates its state every 1 to 30 s depending on the level of vehicular congestion on the network. A highly congested area in the network causes the vehicles to move slowly and hence the vehicle positions are approximately the same within small time intervals (thus the update interval is set at a maximum of 30 s). Free-flow areas in the network with no congestion result in vehicles moving fast and hence vehicle positions accordingly change quickly (thus the update interval is 1 s).

Vehicle position updates in the spatial index can be categorized as local or nonlocal. Any update involves determining the old cell number of the vehicle from the inverse index data structure and the new cell number using the current vehicle position. If the old and new cell numbers are the same, the update is local and simply involves updating the vehicle position (*x* and *y* coordinates). If the old and new cell numbers are different, then the update is nonlocal. The nonlocal update requires deleting the vehicle from the old cell’s list of vehicles and inserting it into the new cell’s list of vehicles. The vehicle information is deleted from the old cell’s list by replacing it with the last vehicle in the cell’s list. The last vehicle’s information in the inverse index is then updated with its new place in the cell’s list. When inserting a vehicle into a cell’s list, it is always added at the end of the list.

To update the vehicle position, we first find the vehicle’s corresponding cell (as described above in Equation (12)) and then linearly search the list of that cell to find the required vehicle information and update its position information. In a large-scale network, the update process is very frequent and will be time-consuming because of the linear search done at each update. In order to improve the performance of the update process, we utilize another data structure which stores the cell each vehicle belongs to and the vehicle’s offset in the list of that cell. In this way, we can update the vehicle position in just two lookups: first, look up the cell number that the vehicle belongs to and then the location of the vehicle in that cell’s list. In this way, we improved the update process from O(k), where *k* is the number of elements in a cell’s list, which could be very large in a large network, to O(1) for any network size.

### 5.3. Modified Analytical Communication Model

In the original analytical communication model, we identified four limitations. First, the model assumed that the vehicles are distributed on a linear highway segment, which is just a small portion of real-life road networks. Second, the vehicles are distributed with a fixed density (μ), which is not the case in real-world scenarios. Third, the inter-vehicle distances are the same and equal to 1/μ, which is not a realistic assumption given the dynamic behavior of vehicles. Fourth, the inter-vehicle distances are considered on the linear axis only, i.e., spaces in front of and behind a vehicle, given the road network structure assumed in the model (a segment of a linear highway). Extending the analytical communication model to allow communication in a realistic road network, we had to address the four limitations described above. We modified the original analytical communication model implementation to capture different mobility patterns, not just highways but any road network structure (or a city layout). We calculated vehicle density dynamically based on instantaneous vehicle locations within the traffic simulator in contrast to the state-of-the-practice implementation that assumes a fixed user-entered traffic density. In this way, we neither assume any vehicle’s distribution on the road (which is dynamic and unknown) nor that the inter-vehicle spacing is fixed or equal. The vehicle dynamics logic implemented in the INTEGRATION simulator governs the vehicle movements and thus controls the inter-vehicle spacings. Finally, the inter-vehicle spacing is considered in all directions around a vehicle using a Euclidean coordinate system. However, we approximate the vehicle density calculations in a linear system, as will be described in the next section. We also improved and sped up the calculations in order to support large-scale road network simulations.

#### Dynamic Density Calculation

Vehicle density is an important factor in the calculation of the probability of packet collision at the receiving vehicle caused by interfering vehicles. Thus, we base our vehicle density calculation on the receiving vehicle’s perspective. First, for each receiving vehicle identified for a given transmitting vehicle, we query the vehicle position database to find the vehicles that exist around the receiving vehicle within a specific range (1000 m in our case following the analytical model). The result of the query is the set of interfering vehicles. We then calculate the distance between the receiving vehicle and each of the identified interfering vehicles at that instant *t* using the vehicle position information. Then, we calculate the vehicles’ density with respect to the receiving vehicle as the inverse of the average distance between the receiving vehicle and the interfering vehicles. Equation (15) shows how the vehicle density is calculated:

(15) $$\mu =\frac{N}{\sum_{i=1}^{N}d_{i,r}},$$

where *N* is the number of interfering vehicles with respect to a receiving vehicle *r* and $d_{i,r}$ is the distance between the interfering vehicle *i* and the receiving vehicle *r*.

In large-scale simulations, the vehicle density calculations are computationally intensive. We can approximate the vehicle density calculations to be from the transmitting vehicle’s perspective instead of the receiving vehicle. In this way, the vehicle density is calculated once per sender vehicle and used for the PDR calculations of all the receiving vehicles of that sender vehicle instead of using a separate vehicle density for each receiving vehicle. This approximation did not affect the accuracy of the PDR calculations; however, it sped up its computational time significantly. A justification for this behavior could be the locality of vehicle positions where vehicles near each other in a specific region of the road network tend to have similar PDR calculations. The spatial locality of PDR calculations allowed for more improvement and sped up the computational time, as will be detailed in Section 5.5.

### 5.4. Bidirectional Dynamic Coupling

Incorporating the analytical communication model into the INTEGRATION traffic simulator resulted in a scalable, closed-loop, bidirectionally coupled mobility-communication modeling framework that allowed for mobility to affect communication and alternatively for communication to affect mobility.

In the first method of coupling (from mobility to communication), the communication component can query the vehicles’ positions at any time instance during the simulation through the vehicle database index component we introduced into the INTEGRATION simulator as discussed earlier in Section 5.2. In this way, mobility patterns affect the communication behavior. Congested road networks would mean a high probability of communication packet interference. Thus, we can test the effect of mobility patterns on the performance of different congestion control approaches. In the other way of coupling (from communication to mobility), the traffic simulator can enable and use different communication applications (e.g., Basic Safety Messages, left-turn assist, intersection movement assist, etc.). The communication application sends and receives packets to provide information for each CV, allowing better decision-making and thus affecting the mobility pattern of vehicles in the network.

The accuracy and the computational complexity of the vehicle position updates and the PDR calculations are controlled by the update interval parameter. Ideally, if we have infinite computing resources, we could update the vehicle positions at an interval equal to the packet generation rate (i.e., λ = 10 Hz or every 0.1 s) and thus could do the PDR calculations at the same interval with maximum accuracy. The trade-off between accuracy and computational time is controlled by the parameter responsible for the frequency of vehicle position updates and thus PDR calculations. When the road network is congested, the vehicles are moving slowly and thus their positions are not changing rapidly. Thus, we can decrease the update frequency of the vehicle positions and do not need to calculate the PDRs. Instead, we use the last recent PDR calculations, which saves computational time but may affect the accuracy of the PDR calculations. When the road network is not congested, the vehicle positions change rapidly, leading to more frequent position updates and thus more frequent PDR calculations, which is computationally intensive but more accurate. To balance the trade-off between time and accuracy, we developed a formula that computes the update interval used in the PDR calculations based on the new positions of the vehicles that are currently in the network as shown in Equation (16):

(16) $$z_{f}=\left\{\begin{array}{cc} 10 & z\leq 10\\ z & 10<z<300\\ 300 & z\geq 300 \end{array}\right.$$

where $z_{f}$ is the bounded update interval and

(17) $$z=\frac{C_{pop}}{C}\times V$$

where *z* is the time to wait to update the PDR calculations based on the new position of the vehicles and is measured in deciseconds, $C_{pop}$ is the number of cells in the grid that are populated with vehicles, *C* is the total number of cells in the grid, and *V* is the number of vehicles that are currently en route in the road network at the time of calculations.

The update frequency $z_{f}$ is bounded between 10 and 300 ds, i.e., the PDR calculations will be updated at least every second and at most every 30 s.

The variable *z* varies as a function of the congestion level of the road network by finding the percentage of the grid ($\frac{C_{pop}}{C}$) that is populated with all the vehicles in the road network (*V*).

### 5.5. Large-Scale Implementation

Investigation showed that the bulk of computations were associated with the PDR collision error computation. Among the variables included in the PDR calculations, only the distance between vehicles and the vehicle density depend on the traffic simulator and are computed each time the calculation is done. All calculation that are not dependent on the vehicle density or inter-vehicle distance ($t_{x}-r_{x}$ and $t_{x}-i_{x}$) are precomputed. They were calculated based on a predefined binning of the distances at the start of the simulation. During the simulation, these predefined calculations are looked up using the current $t_{x}-r_{x}$ and $t_{x}-i_{x}$ distances computed by the traffic simulator. In this way, we save computational time associated with computations every Δ*t*, thus reducing computational redundancy, i.e., the calculation for certain distances and/or densities is performed once, stored, and then looked up every time the same distance and/or density is encountered during the simulation. Modifications were made to further enhance the scalability of the model by computing the PDR per cell rather than per vehicle.

### 5.6. Spatial-Temporal Analysis

The model also computes the spatiotemporal variation of the PDR, which allows us to identify communication holes and locations that need further attention. We show the spatial spread of the PDR across the network during the first hour and during the whole simulation. Using the grid cell index allowed us to perform the spatial temporal analysis of the PDR where we can see the performance of different areas of the network (cells) at any point in time during the simulation.

## 6. Results and Discussion

We validated our implementation of the analytical C-V2X model against the original MATLAB implementation provided in [3] and produced the same results.

We then tested the effect of incorporating the analytical model in the INTEGRATION traffic simulator. We used two road networks for testing: an artificial network called QNET to represent a small-scale experiment and a real-world network for downtown Los Angeles (LA) to represent a large-scale network.

### 6.1. QNET Network Layout

**Figure 6:**
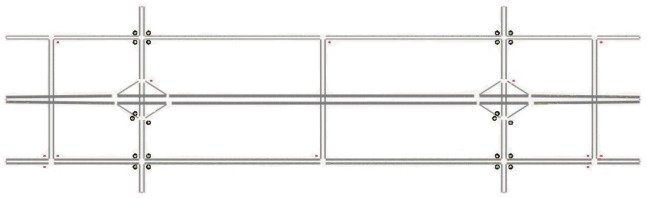
QNET network layout.

The QNET road network is a 3.5-km by 1.5-km area with mainly two horizontal freeway roads with a free-flow speed of 110 km/h and arterial roads with a free-flow speed of 60 km/h. The freeways have three lanes in each direction with one-lane on-ramps and off-ramps. The arterial roads are composed of two lanes per direction. The QNET consists of 32 nodes, 68 links, and 46 Origin Destination (OD) demand entries. The road network has six traffic controls including stop signs and traffic signals. Figure 6 shows the layout of the QNET road network.

The road network is loaded with 4700 vehicles in half an hour and then the simulation is run for an hour to allow all vehicles to clear the network.

### 6.2. QNET Results

**Figure 7:**
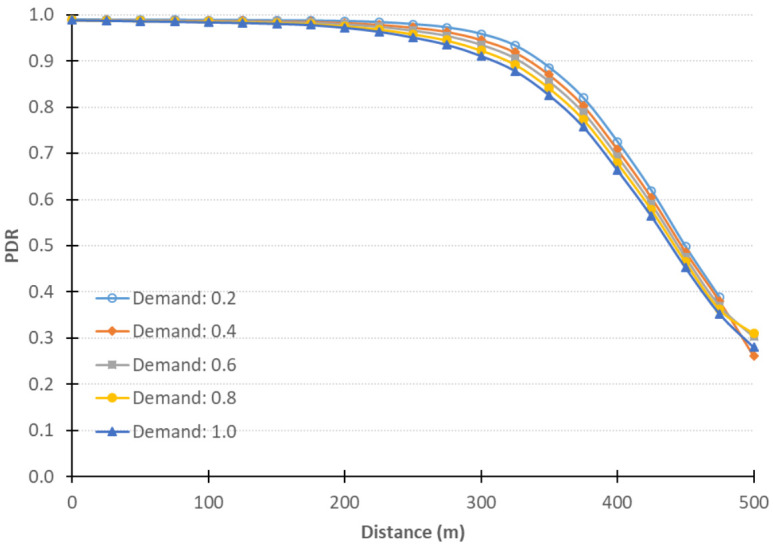
QNET results using different loading percentages.

We tested the QNET road network with different demand levels and with different CV Level of Market Penetration (LMP)s. The original demand is to load 4700 vehicles in half an hour. We tested the performance of the integrated simulator with different levels of demand, namely 20%,40%,60%,80%,and100% of the original demand, in order to show the effect of the level of congestion on the integrated system performance. Figure 7 shows the PDR against different distances at the different levels of demand in the QNET road network. As can be seen, as the demand level increases, the PDR decreases, which confirms that, as the network gets more congested, more packets are lost.

**Figure 8:**
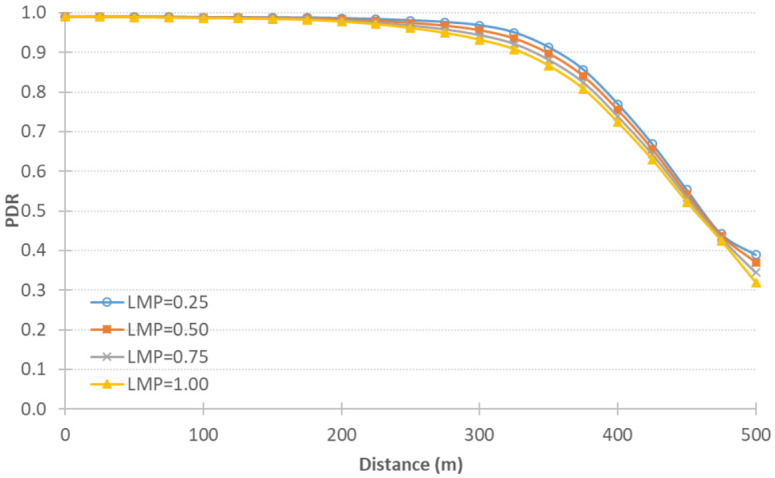
QNET results using different LMPs.

We also tested the integrated system for different CV LMPs, where not all vehicles in the network have connectivity capabilities. We tested with 100% demand while varying the LMP as 25%, 50%, 75%, and 100%. Figure 8 shows the PDR against different distances at the different LMPs. The results confirm that, as LMP increases, the more congested the network is, and thus the more packets are lost.

### 6.3. Downtown LA Network Layout

We tested our system on a real-world, large-scale road network. We used the LA downtown road network using calibrated traffic demand loads. To come up with the OD demands, we used the QueensOD software. The model was constructed and calibrated to meet the actual conditions as described in detail in [24]. The skeleton network was constructed using HERE Geographic Information System (GIS) data. The calibration of the network supply parameters entailed calibrating the four link-specific parameters used in the Van Aerde fundamental diagram:

- Free-flow speed: HERE GIS files using speed class
- Speed-at-capacity: empirical data
- 80% free-flow speed
- Base saturation flow rate: Highway Capacity Manual

We used the HERE Shapefile function class to define the different roadway classes. The jam density was based on a typical vehicle length and assumed to be 180 veh/km/lane.

The traffic demand was calibrated using the QueensOD software [25], which computes the most likely static traffic assignment and origin–destination (OD) demand by iteratively minimizing the error between the observed traffic counts obtained from selected loop detectors and the corresponding estimated traffic volume. The traffic count data needed to generate the synthetic OD files were extracted from Caltrans Performance Measurement System (PEMS) raw data, with 10 randomly selected weekday traffic counts for static OD estimation. A planning OD matrix generated using the standard trip generation approach was used as the seed OD matrix. Time-dependent static ODs were estimated and then used to calibrate a dynamic OD matrix using the procedures described in [26]. A total of 20 randomly selected weekday counts were used for model validation and testing. The coefficient of correlation with observed counts ranged between 0.91 and 0.95.

**Figure 9:**
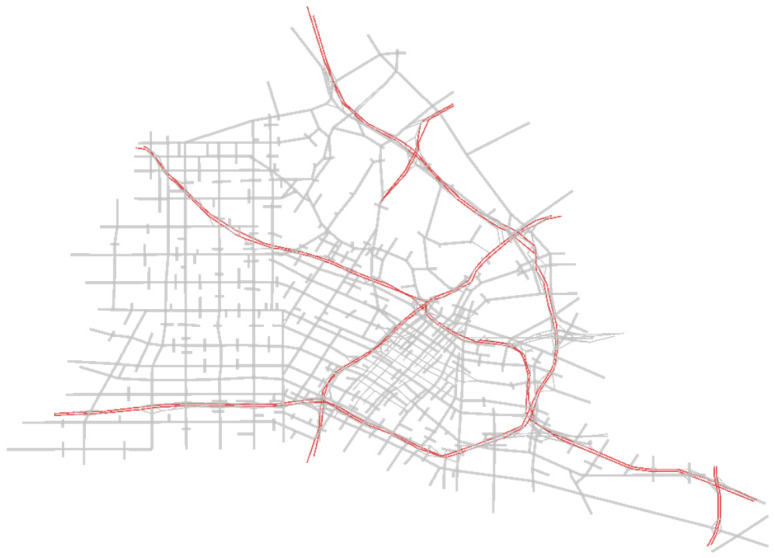
Downtown LA network layout.

Figure 9 shows the layout of the LA downtown road network. The road network is 15 km by 15 km and has 1624 nodes, 3556 links, 457 traffic signals, and 79,384 OD pairs. The road network is loaded in the first hour and simulated for 1.5 h to allow all vehicles to clear the network. A total traffic demand of 145,000 vehicles were simulated with a maximum of 30,000 vehicles concurrently on the network. Three demand levels were considered, namely: 20%, 50%, and 100% of the original peak demand.

### 6.4. Downtown LA Results

**Figure 10:**
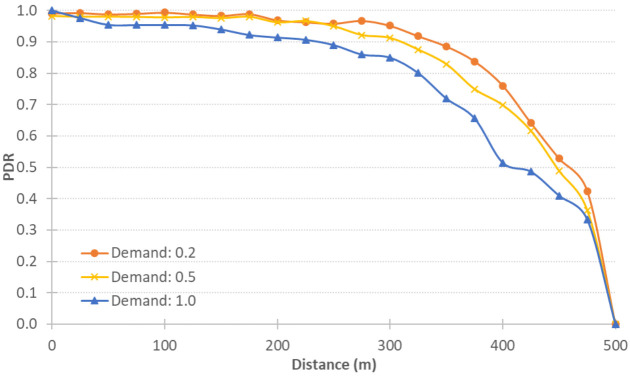
LA results at different demand levels.

We ran the LA simulations at 20%, 50%, and 100% demand levels. All simulations were with 100% LMP. The results shown in Figure 10 confirm the same behavior: as the demand level increases, the more congested the network becomes and the probability of packet collision gets higher, the more packets are lost. In addition, as the distance between the V2V pair increases, the lower the PDR value and thus the more packets that are lost.

**Figure 11:**
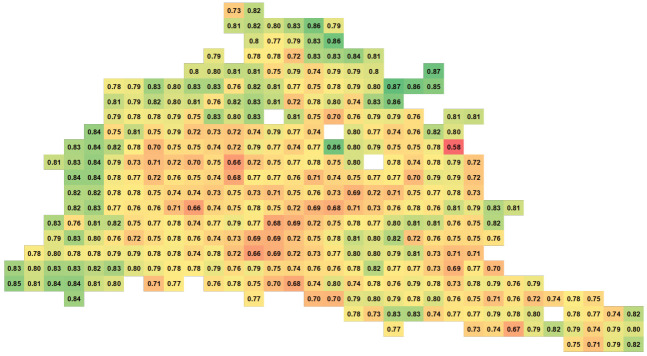
LA spatial analysis of PDR for the entire simulation period. Green cells have high PDR and red cells have low PDR. Yellow cells have in-between PDRs.

**Figure 12:**
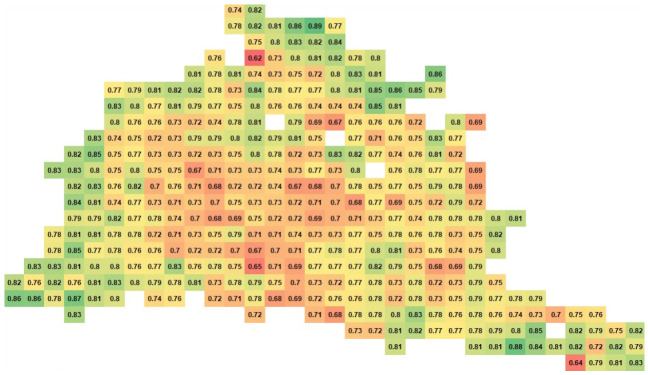
LA spatial analysis of PDR for the first hour of simulation only.

Finally, the vehicle position database, basically the grid cell data structure, allowed us to show the spatial distribution of the PDR in the road network. Thus, we can examine the communication performance in different parts of the road network. Figure 11 and Figure 12 show the spatial distribution of the PDR for the LA road network at the 100% demand level assuming a 100% market level penetration of the connected vehicles during the whole simulation and during the first hour of simulation, respectively. As shown in the figures, the areas with green color indicate high values of PDR and thus good communication performance. The performance degrades in the yellow and red areas. The green areas spread in the edges of the network (the highways), whereas the yellow and red areas are mostly in the inner region of the network (the arterials). In addition, the two figures show the change of the communication behavior across time. Some areas were having bad communication performance and, at the end of the performance, the performance improved. Using our modeling tool, users can perform a spatio-temporal analysis of the performance of the communication module very efficiently for large scale simulations.

## 7. Summary and Conclusions

The paper presents an integrated traffic and communication modeling framework that captures Direct C-V2X communication using a model developed in [3]. Specifically, the authors in [3] developed an analytical formulation that captures four different errors that impact the communication system PDR. The four errors are: half duplex, sensing, propagation, and collision. The first error is caused by the half-duplex nature of the transmission system where a vehicle cannot receive a message while transmitting at the same time. The second error is due to the power of the received signal being below the sensing power threshold at the receiving vehicle. The third error is due to propagation effects, where the received signal-to-noise power ratio is insufficient to correctly decipher the received message. The last error is due to collisions that result from signal interference of two transmitting vehicles, resulting in a low SINR at the recipient. The analytical formulation models the C-V2X communication standard for LTE mode 4, published by 3GPP in release 14 [12], and was validated against OMNET++.

As part of this effort, we enhanced the model to deal with typical transportation networks (extended it beyond linear systems), made the model dynamic, made the model scalable, and incorporated the model into the INTEGRATION microscopic traffic simulation software to develop a fully-integrated dynamic traffic and communication modeling tool. In order to achieve this objective, the software needs to efficiently determine the vehicles within a specified communication range. For a large-scale traffic network, tracking all vehicles within range becomes computationally expensive. Consequently, we developed a framework to track the vehicles’ positions at any time instant during simulation and also to search for vehicles within a specific range efficiently without having to go through all the vehicles. The framework divides the road network into a grid of cells [23] and keeps an index (cell to vehicle mapping) of the set of vehicles that belongs to each cell based on their position in the road network. This index was used to enhance the efficiency of search queries, making the search in O(n) steps where *n* is the number of vehicles in the network. The vehicle positions are updated at a pre-specified time interval. The vehicle is either kept in the same cell or moved into another cell. In the latter case, the vehicle is moved from the old cell and inserted into the new cell list. In order to speed up the process of updating the vehicle positions, another index (vehicle to cell mapping) was developed that stores the cell ID of each vehicle. Using this index, the update process is performed efficiently in O(1) steps.

We ran simulations on the downtown Los Angeles network tracking approximately 145,000 vehicle movements every 0.1 s with up to 30,000 vehicles traveling concurrently on the network. The model was able to run faster than real-time on a regular Windows operating system personal computer generating spatiotemporal PDR estimates.

The unique contributions of this work are (1) we developed a scalable implementation of the analytical communication model [3] that captures packet movement at the millisecond level; (2) we coupled the communication and traffic simulation models in real-time to develop a fully integrated dynamic connected vehicle modeling tool; and (3) we developed scalable approaches that adjust the frequency of model coupling depending on the number of concurrent vehicles in the network. This novel modeling framework provides a breakthrough in the development of urgently needed fully-integrated transportation and communication modeling tool for large-scale testing of Direct C-V2X enabled applications. Further work will use this modeling tool to test various connected vehicle applications to demonstrate the tool’s full functionality.

## Figures and Tables

**Table 1:** Comparison of the proposed system against the Integrated Simulators in literature.

Integrated Simulator	Simulation Scale	Network Simulator	Comm. Standard	Vehicle Positions	Traffic Simulator	Simulator Coupling	Spatial Analysis
Proposed	Large scale	Analytical model	Direct C-V2X	Grid cell and update index	INTEGRATION	Dynamic interval	Yes
VNS [9]	Large scale	NS-3	DSRC (802.11b)	Quad Tree	DIVERT	Fixed interval	No
VEINS [4]	Small scale	OMNET++	IEEE 802.11b	NA	SUMO	Fixed interval	No
Open C-V2X [5]	Small scale	OMNET++	C-V2X	NA	SUMO	Fixed interval	No
Open Source C-V2X [6]	Medium Scale	NS-3	C-V2X	NA	SUMO	Fixed interval	No
VNetIntSim [2,7]	Medium scale	OPNET	IEEE 82.11g	NA	INTEGRATION	Fixed interval	No
Elbery [10,11]	Large scale	Analytical model	DSRC (IEEE 802.11p)	NA	INTEGRATION	Fixed interval	No
IDCVS [8]	Large scale	OMNET++	DSRC (IEEE 802.11p)	NA	SUMO	Fixed interval	Yes

**Table 2:** Road Networks architecture and simulation and execution timing.

	Road Network	Simulation Time	Number of Vehicles	Execution Time
Proposed system	Downtown LA. Area 133 km${}^{2}$. A total of 1624 nodes, 3556 links, and 457 traffic signals	1.8 h	145,000 vehicles with a maximum of 30,000 concurrent vehicles	1.5 h
Elbery [10,11]	Downtown LA. Area 133 km${}^{2}$. 1625 nodes, 3561 links, and 459 traffic signals (42 RSUs)	8.3 h	563,626 vehicles with a maximum of 30,000 concurrent vehicles	8.3 h
VNS [9]	Road network of city of Porto	40 min	130,000 vehicles with a maximum of 15,000 concurrent vehicles	7 h
VNetIntSim [2,7]	An intersection and four zones. Each zone serves as a vehicle origin and destination location. Each road link is 2 km long	Not reported	3000 vehicles with 180 concurrent vehicles	Not reported
Open Source C-V2X [6]	A 100 m × 100 m intersection, and an urban Manhattan grid scenario as used by 3GPP (750 m × 1299 m).	30 s	250 vehicles	Not reported
Open CV2X [5]	A 2700 m six-lane highway section, lane width of 4 m, vehicular speeds of 140 km/h (70 km/h). The inter-vehicle distance of 2.5 s × maximum speed.	Not reported	200 (380) vehicles in the simulation at its most dense stage	Not reported
VEINS [4]	Single-lane Manhattan Grid with intersections spaced 1 km apart. Grid sizes 5 × 5 roads and 16 × 16 roads	Not reported	30 and 1000 vehicles	Not reported

## Data Availability

Not applicable.

## Abbreviations

The following abbreviations are used in this manuscript:

PDR	Packet Delivery Ratio
C-V2X	Cellular-Vehicle to everything
DSRC	Dedicated Short Range Communication
VNS	Vehicular Network Simulator
LMP	Level of Market Penetration
CV	Connected Vehicle
OD	Origin Destination
